# Observations on the Original Malformations and Total Want of Eyes

**Published:** 1834-04-01

**Authors:** 


### VIII.

**BEOBACHTUNGEN URSPRUNGLICHER BILDUNGSFEHLER UND GAENZLICHEN MANGELS DER AUGEN, BEI MENSCHEN, UND THIEREN, Von *B. W. Seiler*. Folio, pp. 64. Dresden.**

**OBSERVATIONS ON THE ORIGINAL MALFORMATIONS AND TOTAL WANT OF THE EYES, IN MAN AND IN THE LOWER ANIMALS.**

(FIRST ARTICLE.)

IT is a striking feature of difference between the medical literature of the last quarter of a century in Germany, and that in Britain during the same period, that while a new science, we mean that of embryology, may be said to have sprung up among, and certainly to have been first properly appreciated and studiously investigated by, our Continental brethren, it should have yet scarcely drawn the attention of a single anatomist in this country, with the exception of the very few who are in the habit of perusing German publications; and even among them, although it may have been admired and approved of in the quiet lucubrations of their own minds, and occasionally, perhaps, discussed at conversational meetings, not one has stepped forward into the arena of public authorship to adduce a single discovery, or to challenge a portion of those laurels which have been so honourably won by Meckel, Walther, Rudolphi, Baer, Burdach, and a host of others throughout Germany. These are proud names for one country to boast of, and if we have not associated with them any of the French school, let it not be supposed that we are actuated by any feelings of envy, or of national rivalry, in so doing; for very far is it from our intention to undervalue, or in any attempt to gainsay, the merit which justly belongs to it. The labours of Geoffroy St. Hilaire, Breschet, Serres, Velpeau, &c. although inferior in eminence to those of their Trans-Rhenal competitors, are worthy of all applause, and of our own most cordial imitation. The English, and not the French, have been the remiss workmen in this vineyard of knowledge; to the shame of the former be it said, they have not begun to lay even the foundation of this building, while the latter have raised it half-way up. But so it has too generally been with our countrymen; how rarely have they ever had the merit of primarily originating any new branch of enquiry! how seldom have they been the first to launch their skiff upon an unknown sea! Perhaps some will answer that it is fully as well, as the result has shewn on more occasions than one, and that if we enquire what is the cause of this national backwardness, the solution will not be found a very difficult task.

A genius for speculation is assuredly not the moving spring of the British character; but, to compensate for this defect, the judging and discriminating powers of the people are far before their talent for invention. The sciences of astronomy, of navigation, of mechanics and of agriculture, and the practical application of their laws and principles, may be adduced as fit examples to illustrate the position we have laid down. To these sciences and arts we may probably add, most of the branches of medicine (using that word in its widest acceptation); for we believe that it is very generally admitted that there is more practical sagacity, and more professional dexterity and usefulness, among the British than among any of the Continental physicians; but when we have claimed this merit, we must not dare to go further—we are decidedly inferior as physiologists, as botanists, and as zoologists; our attention having been directed rather to the every-day occurrences of life, than to the pursuit of less immediate, but certainly of more philosophic researches; and verily to each has been its reward; while the fame and glory of discoveries belong to others, ours has been the no mean praise of testing and sifting these discoveries, of ascertaining their usefulness, and of applying them to the general welfare of mankind. We have been induced to make these remarks, because the hope is strong in our breasts, that the energies of the English mind may soon be directed to the examination of embryological studies. Perhaps there is not a branch of medical enquiry more worthy of the attention of a philosophical mind, than the science of the development of animals; or one which will repay the ingenuous mind with more striking and more beautiful truths. Can we fail to be struck with wonder when told that a being like man, or any of the higher creatures, has, during its embryotic life, passed successively though various grades of formation, the more simple being formed at first, and these allying it to the organization of the humbler animals, and the more and more complex gradually following each other, as the being assumes successively the types of structure which characterise the tribes of aqueous, amphibious, and aërial vertebrates? The very mention of this fact is surely enough to stimulate the curiosity of every one; and it opens up a new and fresh field for philosophic enquiry and speculation, which, while it captivates our fancy by unexpected discoveries, impresses our minds with a more sublime admiration of the infinite wonders of the creating God. The operations of His hand are found to be ever simple, when rightly understood; and even when there seems to be the most irreconcileable disharmony in them, we have but to advance a step or two, and we discover that the chain is unbroken—that there is no sudden or abrupt transition from one link to another, but that all is beauteously connected together, and that as it was contrived by one mind, and as it proceeded from one hand at first, it still bears the impress of one uniformly-pervading character. It was long supposed that animal monsters were often mere rude and formless lumps—that they exhibited no marks of special design, but that they had arisen from some fortuitous and confused agglutination of parts. But such chance work is seldom the case, even with inorganic products, and is never so with any organized creature. A monster, however deformed, will be found quite as organized a being (we do not say as highly) as the perfect animal; it may not, indeed, be capable of independent existence, and it can never, perhaps, propagate itself, but still it is a living thing; and from this mere circumstance alone, of having been impressed with a vital principle, we shall find that we may be warranted in inferring that it has a place somewhere in the zoological series. This position may be expressed more distinctly in the following terms:—"Almost every congenital anomaly or malformation, occurring in a higher animal, has a resemblance to, and has a congeneral type in, the normal structure of some of the lower animals." On another occasion, and that occasion shall not be long delayed, we promise to return to the illustration of this most curious subject; at present, we can only advert to it in connexion with another topic, most intimately associated with it, and which, indeed, cannot well be separated; we allude to the fact, that every mammal embryo has worn, as it were, different, but regularly successive, forms of organization, at different periods of its uterine existence, and that one of the early forms of an organ or part may remain much longer than is normal, and thus the ulterior development be arrested, so that, at the time of birth, a state of parts is found, unnatural indeed, to a fœtus of nine months, but strictly natural to one of three or four months. An example or two will satisfactorily illustrate our meaning. A child is born having the head distended with water, and the brain seemingly quite shrivelled away; but the real truth may be, that the brain, that is the encephalic medullary substance, was never present. We must be wrong, therefore, in supposing that it has become shrivelled from absorption, in consequence of the large quantity of fluid contained within; and the correct rationale of this case may in all probability be discovered to be, that the ulterior development of the part had been stopped or arrested at an early period of its embryotic existence, and that, in place of the complex mechanism of the cerebrum and cerebellum being formed as ought to have been, a condition of the part is left behind, corresponding to the period at which the arrest of development took place. Now if we continue our inquiries, and endeavour to ascertain the different appearances which the encephalon exhibits at different periods after impregnation, we shall soon find that, in the very young embryo, the place of the brain is occupied with a mere vesicle, full of limpid serum—that this vesicle, in course of time, begins to present a division into different parts, and that it is only at a subsequent period that we can discover the slightest traces of medullary matter within. Here, then, we have a state of parts analogous, at least in some respects, to the state which many hydrocephalic monsters exhibit at the time of birth. Equally illustrative of the same doctrine are all the examples of spina bifida, of concreted fingers and toes, of the persistence of the membrana pupillaris, and so forth; but it must be quite unnecessary to multiply proofs now, when our readers will find, on the perusal of this paper, that almost every congenital anomaly of the organ of sight has its type in the normal condition of the part, at some epoch or another of the embryo's existence. The work of Dr. Seiler is devoted exclusively to the investigation of ophthalmic malformations, and from the very great erudition which it displays, we predict that it will be received by all as a most valuable monograph on one theme of embryology.

The first section is occupied with the consideration of the general malformations, or of those which affect the entire organ; the second gives a description of the malformations of its special parts or structures: In treating of the first class, or of the general malformations, we shall allude to them as they affect the number, situation, size, form, and the very existence of the eyeballs, mentioning, under each head, the most interesting embryological changes which the organ undergoes, up to the time of its complete maturity.

### GENERAL MALFORMATIONS.

### I. Number of Eyes.

*Supernumerary.* There is no well confirmed or trustworthy example on record of more than two eyes having ever been found in a head which was truly single; that is, where there were no traces of the melting together of two heads discoverable. Many authors indeed have told us of human fœtuses which had three or four eyes; but these were in reality bicephalous monsters, and were no doubt provided with two noses, four ears, a double mouth, and so forth, or at least with rudiments of such anomalies.

*Defective.* Rudolphi has detailed the particulars of a genuine example of monophthalmia, in which, one eye was quite awanting, while the other was present. The child was otherwise healthy, born at the full time, and lived for sixteen hours. There was no trace of the right eye, or of any of its appendages, such as the eyebrow, eyelids, muscles, and nerves. Nothing could be seen in its place, but a shallow furrow, and the orbitar plates of the frontal and of the upper maxillary bones had so grown together, that the skin of the forehead passed almost quite smooth and level down to the cheek. But other parts also were deficient; thus there was neither a right olfactory nerve, nor a right optic nerve to be found; the left optic was regular; and at the usual place of the meeting of the two nerves, it gave off a small process, which terminated in an empty sheath, continuous with the dura mater. The third, fourth and sixth cerebral nerves on the right side were wanting; on the left they existed as usual. The other cerebral nerves were present on both sides. The right optic thalamus was of a pyriform shape and terminated in a thin, short point, which was continuous with the commencement of the posterior lobe of the brain, or with the prolongation of the cornu ammonis on that side. The right lateral ventricle was smaller than the left: the tenia semicircularis was wanting, and the cornu ammonis was very indistinct. Water was found in the fifth ventricle; and the third was larger than usual. The right anterior crus of the fornix was wanting, and also the right corpus mamillare.

Professor Walther has recorded a case similar in many respects to the preceding; in it the left eye was deficient; the bony orbit was very small; and the lids were almost quite shrivelled away.

Otto found a similar anomaly in a calf, which he examined.

Such cases as these we have alluded to ought to be carefully distinguished from another sort of "monophthalmus," or as St. Hilaire has better called it, "monopsia;" we mean that in which the existing eye is situated in the median line; the former may be denominated perfect; the latter imperfect, or Cyclopic. Now the difference in these two forms is important; for in the one, the existing eye may be quite normal in its formation; but in all the cases of the other, which have been attentively examined, there have been more or less distinct traces of a second eye (such as double cornea, double lens, double iris, &c.); even in such an example as Haller describes, where neither the cornea, sclerotic, choroid, nor lens exhibited any marks of duplicity, except their unusual size, although the upper eye-lid was to all appearance compound. There thus seems to be an effort in the formative principle to the construction of two eyes, or at least of a duplicate of some of its structures. The consideration therefore of the Cyclopic variety of monopsia belongs rather to the anomalies of the position than of the number of the organs.

### II. Position of the Eyes.

The anomalies in this respect are very various; the eyes may be situated too high or too low in the face; too near to, or too far apart from each other; unusually sunk, or over-prominent; or the direction of their axes may be changed from the normal line.

In anencephalous, microcephalous, and hydrocephalic monsters, an extraordinary projection of the eyes is not uncommon; and this may be occasioned either by some irregularity in the normal direction of the bony walls of the orbit or by an imperfect development of one or of several of the bones, which enter into its formation. In some rare cases the protrusion of the eye amounts to "exophthalmia congenita," or "ophthalmoptosis."

The stories which we read of in the writings of Pliny and of many later authors, about their having seen monsters, whose eyes were placed on the breast, in the axilla, on the shoulders, or on the back of the occiput, are quite fanciful and untrue; in all probability the fœtuses were acephalous, and wanted the cervical vertebræ in the first set of cases; and in the latter, or occipital-eyed monsters, there might be two heads confusedly joined together; or lastly, what were taken for eyes, were really not so, and only somewhat like to these organs. To descant upon this subject would be an idle loss of time; we shall therefore proceed to the consideration of some malformations, which are more common, and ought therefore to be more attentively examined. Every one knows that the eyes are sometimes situated nearer to each other than is natural; and that at other times they are abnormally apart. There are various degrees of the former of these malformations; the extreme being that wherein the two orbits are almost quite melted, as it were, into each other, the eye-balls however remaining distinct; and which may be said to prepare the way for "monophthalmus," or imperfect cyclopia." The anatomical investigation of such examples, shews that there is an imperfect development of the frontal processes of the upper jaw, of the æthmoid, lachrymal, and of the nasal bones. Meckel, in his manual of pathological anatomy, has ascribed every example of monophthalmus, as well as of monopodia, or one-footedness, to the concreting or melting together of the parts, which are naturally double; but more lately he has modified this doctrine, and admitted, that these monstrosities may arise from the development of the organs being somehow or another impeded or arrested. Still more recently that acute observer Huschke has come to the conclusion, from multiplied investigations of the very early embryo chick, that monophthalmus ought to be regarded, not as a colliquation of the two eyes, but rather as a continued or over-prolonged existence of a natural state of parts. We are inclined to subscribe to this doctrine of Huschke; but at the same time we acknowledge that the hypothesis which supposes that the most perfect, or complete form of cyclopia, or of genuine monophthalmus, corresponds with a still earlier period of the normal formation of the eye, requires before it can be received, the confirmation of numerous experiments, especially on the embryos of mammiferous animals: As the extreme softness and delicacy of the structures of the early chick render any examination of them most difficult and uncertain, it will be a great achievement of some future anatomist, to discover a method how to investigate a mammal embryo, at that period of its existence when it is supposed to correspond or be analogous to the young chick on the first day of incubation. Notwithstanding numerous trials, our author informs us that he has not hitherto been more fortunate than Harvey, Haller, Bäer, and others, in determining exactly the day when the embryo may first be discovered after impregnation. In the sheep and dog he has never found it before the 19th or 20th day; and at this period we may almost always observe at the sides of the anterior extremity two small black dots, which are the rudiments of the eye-balls, or at least correspond with the position of the sockets. The same appearances Dr. Seiler has seen in a human embryo, only 21 days old.

We have already observed, that the anomaly which we have been describing is very different from the complete, or Cyclopic monophthalmus. The two kinds may so far agree, that a single eye lies in one simple socket, but the number and form of the parts do not ever quite correspond in each. Both of these anomalies in the formation of the eye are very commonly associated with traces of arrested development, in several of the adjoining structures; especially in the bones of the face, and also of the cranium; hydrocephalus is a frequent accompaniment; and even some of the nerves may be found altogether awanting on dissection. The more distant parts of the body may be simultaneously affected; thus there may be spina bifida, umbilical hernia, uterus bicornis, concretion of the two kidneys, and so forth.

The cyclopia is by no means unfrequently met with in the fœtuses of some mammal animals, especially in the sheep and swine; more rarely in human fœtuses; and still more rarely in the next class of animals, the birds: such at least is the order of frequency, as hitherto ascertained. Those who are anxious to obtain moie extended information on this very interesting subject, should consult the manuals of pathological anatomy, by Voigtel, Meckel, Otto, &c., and the seperate memoirs of Tiedeman and Huschke.

### III. Size of the Eyes.

In judging of the size of this organ we must not forget to remark attentively the degree of its projection from the socket; for should this be unusually small, while the eye is of ordinary dimensions, an observer might be led to believe that he had met with a case of enlargement of the eyeball, or of megalophthalmus. It is only necessary to allude to this source of error, to prevent our readers from committing it. In the genuine anomaly, not only is the organ apparently, but it is actually and intrinsically magnified, sometimes in all its component structures, at other times only in some of them, as the vitreous humor, cornea, &c. There is very generally an accompanying disease, or malformation, such as cyclopia, hydrocephalus, or anencephalus, in cases of megalophthalmus.

Our author has figured a well-marked example of this anomaly in a fœtus; the longitudinal diameter, from the superior to the inferior rectus muscle, measured half an inch; the transverse one was the same; and the antero-posterior diameter, or axis of the eye, was seven lines.

In congenital hydrocephalus the vitreous humor often retains its primitive fluidity; and as the quantity of this may go on increasing, the eye-ball speedily acquires an enlarged size. In one case detailed by our author, in which the optic nerve was quite awanting, and only its neurilema remained, this sheath was found distended with a fluid, in every respect similar to that contained simultaneously within the dura mater of the brain; a fact, among others, which has led Huschke to maintain that a passage or canal exists primitively between the lateral ventricles to the eyes; that there these canals expend themselves into globular sacs in order to contain the humors, and to afford surfaces for the extension of the retinæ. He thus considers the ocular humors as part of the fluid which so abundantly fills up the cells of the very young embryonic brain, and which in the after age collects into a few drops within certain definite cavities, denominated the ventricles. In confirmation of these doctrines, Huschke alludes to the state of the vitreous humor in the lower vertebrated animals, in which it has nearly the same consistence and chemical properties as the cerebral fluid. We may therefore adduce this as one among a host of other examples, proving the identity of a malformation in a higher animal, with a normal formation in a lower.

The opposite anomaly to that which we have been describing is "microphthalmus," in which the eye or eyes are unusually small. Beer, in 1813, was the first to notice distinctly this affection; and subsequently it has been illustrated by Pœnitz, Gescheidt, and others. One case which has been narrated by Pœnitz was observed in an infant four weeks old; the eyelids were normally formed, but the eyeballs, and especially the corneæ were much too small; the outer margin of the left cornea was not uniformly circular, but irregular and notched. The iris seemed to lie on the cornea, and the pupil was not larger than a fly's head. The right cornea was larger by one half than the left, and was so opaque that the boundaries between it and the sclerotic coat were not manifest; the latter seemed to be prolonged or continued in striæ over the former, so that no part of this cornea was transparent. The right pupil was as diminutive as the left one. This case is especially valuable as it occurred in a living child, which continued to exist for some time, and in which any change after birth might therefore be observed. Now the very interesting fact was observed, that by the twelfth week the corneæ were not only considerably larger than in the fourth, but they had become also clearer, more transparent, and more distinctly separated from the sclerotic. The pupils too were rounder and blacker than before.

Fisher has briefly related two cases of microphthalmus; one occurred in a boy; the left eye was wanting; and the cornea of the right one was not larger than a small pea, and utterly useless;—the other case occurred in a girl, who also wanted the left eye, and in whom the right cornea was exceedingly defective. In the case of an infant, six weeks old, seen and described by Weller, Gescheidt, and our author, both eyeballs were abnormally small; the irides were altogether awanting in their inferior third, and the other two-thirds were very small. With these exceptions the eyeballs were not formed amiss. When the child was three years old, the right eye was one-fourth smaller in all its dimensions than the left. The fissure between the right eyelids was extremely short, and the upper lid hung down over the lower one, so that the eye could not be examined without difficulty.

In both eyes there was a coloboma, or partial deficiency of the iris; the convexity of the left cornea was normal, but the under segment of the right cornea, was not only less projecting, but also smaller than the superior one.

In Meckel's Archives of Anatomy and Physiology for 1830, we have the paticulars of a case which was observed in a girl, nine years of age; both eyes were at least one-half too small; the different parts of the right one seemed to be quite normally formed; but the left lens was opaque. The eyelids could be separated but a very little way from each other. A younger child of the same family presented similar defects. In both, the sensibility to light was almost or entirely gone. Escher mentions a case of microphthalmus which he saw in a woman 41 years of age. In her the right eye was and had been from infancy one half smaller than the left; its convexity was normal, and it lay very deep in the socket; the eyelids were almost always closed; but on separating them, the cornea was found transparent, the iris of a yellowish grey colour, but partially awanting, and the lens or its capsule was opaque. The right eye was altogether normal.

Schoen has related in Ammon's Journal of Ophthalmology, an interesting example of this affection which he observed in a well-formed female infant, ten days old. The eyelids of both eyes were concave outwardly, being drawn into the sockets. On separating those of the right side, as far as they could be, he saw, deep hid in the socket, a whitish red body, not larger than a pea, having a small black speck in the middle, (a rudiment of the iris and pupil,) moving from one side to another. The almost empty socket appeared to be lined with a loose red-coloured membrane, which was continued over the inner surfaces of the two lids. The left eye-ball was considerably larger than the right one, but still much smaller than natural; and its cornea was flattened, and not easily distinguishable at the margin from the sclerotic; although it was transparent, neither an iris nor a pupil could be distinctly seen through it. When this child was six months old, it was found that the right eye remained nearly in "statu quo;" but the left one had increased in size;—no iris however could yet be discovered. There seemed to be a complete insensibility to light. Another very curious example is given by the author who has communicated the preceding. The girl was seven years of age when he saw her;—the left eyelids were kept always closed, and the cleft between them was a quarter of an inch shorter on this side than on the other. When opened, he observed a rudimentary eyeball, surrounded with cellular tissue and fat, in the middle of and deep within the socket; it was of a whitish colour, and moved about freely and regularly; the cornea was transparent, but too flat; the dull-blue iris was motionless; the pupil was large but defective at its lower part, or, to use a Celsian phrase, was slightly colobomatous. Very little change had taken place in either eye since infancy; and the girl had been always quite blind.

Numerous examples similar to those now described may be found, no doubt, in every institution for the blind, in this, and in most other countries. As yet indeed little attention has been paid to the very curious, and we may add, very instructive (at least to the philosophic physician and surgeon) subject of congenital malformations in the British dominions; but it is to be hoped that the future will be more rich and promising, and that we shall follow the admirable example of the continental, and more especially of the German anatomists, in investigating the wonders of embryology. No exertion shall be wanting on our part to afford every assistance, whether by publishing the original communications of native enquirers, or by presenting faithful abstracts of the most approved writings of foreign authors. It is to be hoped that the present article will be received as an earnest of the sincerity of our promises.

To return "ad rem;"—our author alludes to the dissection of a case of microphthalmus reported from the blind institution at Dresden. In C. Herzog both eyes were preternaturally small, and the sight had been always very defective. This man died of typhus fever; and upon examining the body after death, the following appearances in the eyes were found. Their form was more lengthened than is usual; the size of the optic nerves was in proportion to that of the balls; the scleroticæ were somewhat abnormally thickened, but otherwise healthy; the choroid coats were equally so; the vitreous humors were transparent, but scanty in quantity; but there were no traces of the lens in either eye;—the crystalline capsules were however thick, yellowish, and, as it were, rumpled on the surface; the coronæ ciliares were indistinct, and the uveæ but imperfectly coloured.

From an attentive consideration of the cases of microphthalmus, especially when these are viewed in connexion with the other signs of arrested development, which are so commonly associated with them, we find numerous proofs in confirmation of the admirable descriptions of the gradual development of the eyes in the embryo, recently published by Ammon and Huschke. Some of the successive steps or stages of the development are yet imperfectly known, and probably may long continue to be so, in consequence of the extreme softness and delicacy of the embryonic structures; but there is good reason to hope, that the gaps in the enquiry may be, in some degree at least, filled up, if all the phenomena of microphthalmus, at different periods of life, from birth to adult age, be well and satisfactorily investigated; more particularly if every change which may take place during the lives of the patients be diligently observed and accurately recorded. On this, as on every other subject of experimental enquiry, there must be a rich accumulation of authenticated facts to form a groundwork for safe deduction; the humblest labourer may contribute his quota; but it remains for a master, like the great Cuvier, to trace the beautiful harmony of the whole. According to Huschke's observations the most anterior and the broadest dent in the "primitive fold" or "dorsal plane" of the young embryo, is the earliest trace of the eye-ball with its socket. The eye, he says, appears originally as a half canal or open hollow on the germinal membrane; and this appearance may be seen in the chick, on the first day of incubation. In the second step of development, the hollow or dimple is converted into a bladder or cell, which looks as if it were formed by a tucking up of the meningeal membrane. The primary parts, or those first developed, are therefore the cavities of the eye-ball, which proceed or are formed from a folding of the outer integuments; they are open at first, and their parietes are connected partly with these and partly with the membranes of the brain. As yet there is no vestige of a crystalline lens; and the only appearance of the vitreous body is a transparent fluid, which, without being contained in any proper membrane, fills up the hollow of the scarce closed eye-ball, and seems to be continuous with that in the cells of the brain, still free from any medullary substance. At first the individual membranes of the eye are quite undistinguishable; and also the muscles and all the other appendages. At a somewhat later period the sclerotic and choroid coats may be made out, and after these the retina. Now if we stop here and compare the preparations which illustrate the arrests of development at different periods, we shall find some which exhibit appearances altogether similar to those described, as to be seen in the chick on the second day of incubation. Thus in one mentioned and figured by Dr. Seiler, the eye is nothing but a membranaceous cyst, which is formed externally by the sclerotic, and on whose inner surface the choroid lies, and which is filled with a transparent fluid, while all the other structures of the organ are quite awanting. The chief difference between the two cases is, that in the embryonic specimen there is an immediate connexion of the cyst with the cerebral membrane, whereas, in the abnormal one of a later date, this connexion is effected by means of a ligamentous or nervous canal, which may be either empty, or which may contain medullary fibres. In order to explain the connexion between the membranes of the eye and those of the brain, and to illustrate the development of the retina and of the vitreous humor, we shall extract a passage from the instructive memoir of Huschke, on the anatomy of the chick in ovo.

"Proceeding from the fore part of the cerebral ventricle, there is a canal, which again expands in the eye, inclosing the retina, and which forms a passage for a part of that fluid, with which at this period the brain-cells are filled. This fluid is subsequently converted into the proper vitreous humor. In the lower animals of the vertebrated division it retains nearly the same consistence and chemical properties as those of the contents of their cerebral ventricles. In the very young embryo this fluid appears not to be contained within any proper membrane—at least such cannot be made out. If one does actually exist, it is no doubt a prolongation from the investing membrane of the ventricles. Admitting therefore that the retina is continued from the medullary walls of the cerebral cavities, and that the vitreous body is analogous to, and indeed derived from the serum within these, we are led to believe that the membrana vitrea corresponds to their epithelium. Many of the phenomena exhibited in cases of congenital hydrocephalus are in unison with, and therefore may be supposed to confirm these ideas. During the early stages of the development of the nervous system there is a large quantity of fluid diffused through every part of its substance; and should an arrest or impediment to the ulterior progress of the development ensue, while the mere process of enlargement or increase of size is going on, a state of parts is exhibited, agreeing in almost every respect with the appearances observed in the congenital disease above-mentioned."

These ingenious views of this acute physiologist are well corroborated by some of the drawings appended to Dr. Seiler's work:—In one, illustrative of a microcephalus monstrosity, we observe the perfectly fluid and transparent serum in the place of the more consistent vitreous body; and in another is given the representation of a canal proceeding from the dura mater, and communicating inwardly with the watery cyst, in which the brain should have been contained, and outwardly with the cavity of the eyeball. This cavity was filled with a clear serum, altogether similar to that in the cerebral bag. No traces of retina were to be seen, probably because the medullary substance had not yet begun to be formed. It is to be remembered that such a state of parts is quite analogous to the condition of the eye and brain in a chick, on the 2d day after incubation.

"The crystalline lens begins to be formed," says Huschke, "in the following manner. When the embryo chick is two days and a few hours old, there appears suddenly a small circle within and concentric with the larger one, which we formerly described, to be the earliest rudimentary trace of the eye. This smaller circle indicates the formation of the lens;—it is clear at its edge, and somewhat opaque in the middle, but it is not cleft at any part, as the outer ring is, and it is perfectly round. The membrana crystallina is first formed, and in the following manner:—A small part of the fine membrane which covers the primitively open eye-dimples or hollows is pressed in, just as a sebaceous cyst is, from the investing layer of skin; the opening thus made becomes gradually narrower, and at length is altogether shut and becomes separated from the dermoid structure whence it was derived. The closing together takes place from before backwards, and so gradually that until about the middle of the third day of incubation, the entrance into the anterior wall of the crystalline capsule can, with the assistance of a magnifying glass, be seen as a dark-coloured spot. It follows, therefore, that the capsule of the lens is a portion of the tegumentary membrane, which becomes reflected inwards, and from which it is afterwards quite detached. The substance of the lens itself is subsequently generated as a secretion from its capsule." Our author (although he admits the startling prima facie unlikelihood of the supposition, that after the cyst of the embryo eyeball has been once closed any part of its walls should become reflected inwards, to form such a tender and delicate structure as the crystalline lens) is inclined to accede to the above explanation given by Huschke. His authority, we are told, stands so high among his countrymen, and his extreme aversion from all precipitate inference is so well known, that we are bound to give credit to his descriptions until they are gainsayed by any competent observer. It appears, therefore, that the vitreous humor is formed some time before the lens; and we find that this result of anatomical investigation accords with some of the anomalies of structure in cases of congenital malformations of the eye. Thus the lens is occasionally awanting, while the humor is present. There is reason however to suppose, that when once the crystalline capsule is completed, the generation of the contained lens is very rapid. The abnormally deep position of the lens in the eyeball, observed sometimes in microphthalmus, arises probably from an arrested development of the vitreous body, which, according to the researches of Ammon, is, up to the third month of conception, very small in bulk compared with that of the lens. In the circumstances of the blueish colour of the sclerotic, the obscurity or dulness of the cornea, and of the indistinct limitation between this membrane and the sclerotic, noticed in many microphthalmous children, we recognise the close analogy which exists between these occurrences and the state of parts in a fœtus about three months old. It is about this period that there is any evident distinction between the cornea and the sclerotica; but still the union of the two remains very close and strong, and cannot indeed be well separated either by maceration or in any other way;—they seem to be, as it were, melted into or amalgamated with each other. At a later period of fœtal life we first observe the iris. Ammon states that he has generally discovered a faint trace of it in a fœtus of about five months. In the complete absence of this curtain, and probably also in the coloboma, or partial deficiency of it, we recognise the manifestations of an arrested development. If we now turn our attention from the eyeball to the eyelids, we shall meet with examples of congenital malformations, which beautifully illustrate the truth of the law now mentioned. But, first of all, it will be useful to borrow Ammon's description of these parts in the human embryo, for the sake of an accurate comparison. "The eyes of a three-months' fœtus are not covered, but only embraced, or, as it were, girdled, with the lids ; at a still earlier period, the balls quite project beyond the surface, but gradually become more and more depressed, or at least more inclosed, than heretofore, being received into the sockets, which hitherto were not formed. Cotemporaneous with the formation of the sockets is the first appearance of eyelids ; these are minute folds of the skin, surrounding and embracing the eyes. The appearance of the eyes at this period is like that of an acorn in its cup. By the end of the third, or in the first half of the fourth month, the lids have so much increased in growth as to fairly cover the eyeball.

In fœtuses aborted at the fifth month, the eyelids are always found closed or glued together, but the one not perfectly in apposition with the other. If the parts be examined while they are quite fresh, it is observed that the gelatinous and very red skin forms processes or prolonged folds over the still very projecting eyeballs—the blue bulbs very distinctly glimmering between these processes. The cicatrix-like chink which lies transversely between them exhibits, under the magnifying glass, two small yellowish striæ, which are afterwards discovered to be the rudiments of the Meibomian glands. Numerous bloodvessels supply the eyelids ; these come partly from the auricular, and partly from the labial and nasal trunks." Our author has had frequent opportunities of confirming the accuracy of the above description, given by Ammon ; and we shall find that, by attending to the different stages of the normal development of the eyelids, we are furnished with a key to the right explanation of many of their congenital malformations, and to a more correct understanding of their minute anatomy. We can thus, for example, ascertain to a certainty, that the conjunctiva is expanded over the whole anterior surface of the eyeball, at first quite loosely, but gradually more and more intimately ; and an unanswerable proof is afforded, at the same time, against the doctrine of Rudolphi and of others, that the conjunctiva is analogous to the epidermis. Its true nature is intermediate between a serous and a mucous membrane ; in the earliest stages of its formation, it would seem to be a shut sac, and only after the inter-palpebral cleft is completed, to become a mucous membrane.

For the purpose of shewing these phenomena, it is necessary to cut away two lateral halves of the lids, so that the ball of the eye is seen from the side ; if this be done, we display a sacciform envelope, whose posterior surface covers the bulb, and whose anterior one is reflected upon the inner surface of the eyelids. By means of it, the eyelids and eyeball are joined together, but its connexion with the latter is much more close than with the former. It contains a transparent watery fluid, which thus prevents any agglutination of the lids with the eyeball. Such is Ammon's account of the early formation of the conjunctiva ; and if the investigations of succeeding enquirers accord with it, we are bound to accede to the conclusion which we have just now mentioned has been arrived at by the celebrated anatomist now mentioned, viz. that the conjunctiva is at first a serous, and only subsequently a mucous membrane.

But to return to the subject of the eyelids ; we have to state that several examples of their arrested development are narrated by Dr. Seiler, and that all these harmonize most beautifully with the descriptions of their early formation in the embryo. The fourth figure of his engraving represents on a large scale, in a young infant, the normal state of the eyelids of a three-months' embryo ; they are short and small, and cover only very imperfectly the extremely projecting ball, which is thus not unlike to an acorn within its cup. But not only have the lids been arrested in their ulterior development, but also some of the other accessory parts of the eye ; thus, the muscles are yet most imperfect and indistinct, for the organ seems to be surrounded merely with a white fibrous covering, just as we find it in a three-months' embryo, and there is no obvious separation into individual bundles. Dr. Seiler takes this opportunity of expressing his dissent from the statement made some years ago, in the Journal der Chirurgie, by his eminent friend Walther, one of the editors, that the eyelids originally, or primitively, adhere to the anterior surface of the ball, and, therefore, that congenital anchyloblepharon is referable to a pre-existent normal condition of the parts. This error has arisen from his having mistaken the delicate sacciform covering, which we have shewn to be the commencement of the conjunctiva, for rudimentary palpebræ; and also from his not distinguishing properly a mere conglutination of the palpebral edges with their actual concretion.

*(To be continued in next Number.)*

